# Formalizing biomedical concepts from textual definitions

**DOI:** 10.1186/s13326-015-0015-3

**Published:** 2015-04-02

**Authors:** Alina Petrova, Yue Ma, George Tsatsaronis, Maria Kissa, Felix Distel, Franz Baader, Michael Schroeder

**Affiliations:** Biotechnology Center, Technische Universität Dresden, Dresden, Germany; Institute of Theoretical Computer Science, Technische Universität Dresden, Dresden, Germany

**Keywords:** Formal definitions, Biomedical ontologies, Relation extraction, SNOMED CT, MeSH

## Abstract

**Background:**

Ontologies play a major role in life sciences, enabling a number of applications, from new data integration to knowledge verification. SNOMED CT is a large medical ontology that is formally defined so that it ensures global consistency and support of complex reasoning tasks. Most biomedical ontologies and taxonomies on the other hand define concepts only textually, without the use of logic. Here, we investigate how to automatically generate formal concept definitions from textual ones. We develop a method that uses machine learning in combination with several types of lexical and semantic features and outputs formal definitions that follow the structure of SNOMED CT concept definitions.

**Results:**

We evaluate our method on three benchmarks and test both the underlying relation extraction component as well as the overall quality of output concept definitions. In addition, we provide an analysis on the following aspects: (1) How do definitions mined from the Web and literature differ from the ones mined from manually created definitions, e.g., MeSH? (2) How do different feature representations, e.g., the restrictions of relations’ domain and range, impact on the generated definition quality?, (3) How do different machine learning algorithms compare to each other for the task of formal definition generation?, and, (4) What is the influence of the learning data size to the task? We discuss all of these settings in detail and show that the suggested approach can achieve success rates of over 90*%*. In addition, the results show that the choice of corpora, lexical features, learning algorithm and data size do not impact the performance as strongly as semantic types do. Semantic types limit the domain and range of a predicted relation, and as long as relations’ domain and range pairs do not overlap, this information is most valuable in formalizing textual definitions.

**Conclusions:**

The analysis presented in this manuscript implies that automated methods can provide a valuable contribution to the formalization of biomedical knowledge, thus paving the way for future applications that go beyond retrieval and into complex reasoning. The method is implemented and accessible to the public from: https://github.com/alifahsyamsiyah/learningDL.

## Introduction

Research in the biomedical domain is characterized by an exponential growth of the published scientific materials, e.g., articles, patents, datasets, technical reports. Handling such a scale of information is a huge challenge, for the purpose of which multiple initiatives have been launched in order to organize biomedical knowledge formally. The use of ontologies is one of the most promising key aspects in this direction that has attracted a lot of interest [1]. An ontology is a complex formal structure that can be decomposed into a set of logical axioms that state different relations between formal concepts. Together the axioms model the state of affairs in a domain. With the advances in *Description Logics* (DL), the process of designing, implementing and maintaining large-scale ontologies has been considerably facilitated [2]. In fact, DL has become the most widely used formalism underlying ontologies. Several well-known biomedical ontologies, such as GALEN [3] or SNOMED CT are based on DL.

With regards to the potential benefits of formal knowledge in biomedical research, there exist already two examples where such a formalization has helped towards knowledge discovery. In the first case, a formal ontology about human anatomy with over 70,000 concepts (FMA) is used to infer potential internal and hidden injuries from injuries that are visible in images [4]. In the second case, the authors show how reasoning over yeast metabolism can generate novel hypotheses [5]. The necessary background knowledge and reasoning framework form a crucial part of a “robot scientist”, which autonomously executes and evaluates experiments in yeast. Thus, formalizing biomedical knowledge can assist important biomedical applications. However, the problem of formalizing the knowledge in the domain is an open problem, since most biomedical ontologies and vocabularies, such as GO, MeSH, OBO, define concepts only informally by text strings. Hence, the main problem is to convert textual definitions into formal representations.

A key step in formalizing knowledge in biomedical domain is to extract formal definitions for biomedical concepts. As explained in [6], “concepts are formally defined in terms of their relationships with other concepts. These “logical definitions” give explicit meaning which a computer can process and query on”. In Table 1, the second row gives an example of a formal definition of a concept Baritosis. The definition reads as follows: “Baritosis is a sort of Pneumoconiosis and relates to another concept Barium_dust via the relation Causative_agent”. The first row of Table 1 is the corresponding textual definition. Indeed, almost all of the existing biomedical formal ontologies, such as OpenGalen, SNOMED CT, FMA, contain only such kind of formal knowledge due to the essence of real practice, though DL theory allows for more expressive representation (e.g. General Concept Inclusions [2]). Thus, in this paper, we focus on learning formal definitions of concepts.

**Table 1:** **Textual and formal definitions of** ***Baritosis***

Textual definition “*Baritosis* is a benign type of *pneumoconiosis*, which	
is caused by long-term exposure to *barium dust*”.	
Formal definition *Baritosis* ⊑*Pneumoconiosis* ⊓	
∃*Causative*_*agent.Barium*_*dust*	

**Figure 1:**
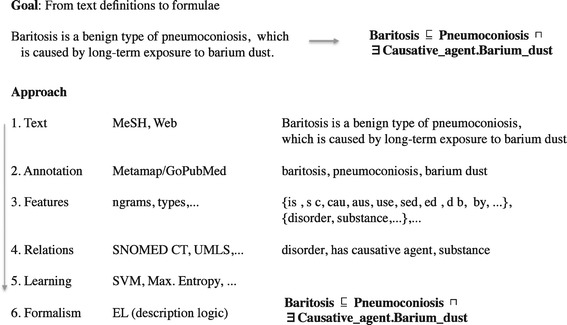
Overview of the main aspects related to automated extraction of formal concepts definitions, via a simple example of the definition of *“Baritosis”*. The figure illustrates an established text mining workflow based on supervised machine learning to address the task. In this work we analyze the impact to the overall performance of the different aspects, namely: modeling (selection of corpora and relations set), feature engineering (selection of lexical and semantic features) and machine learning (selection of classifiers and number of training examples).

Unlike the taxonomy acquisition which seeks to identify parent-child relations in text and is usually based on simple patterns [7], definition generation typically focuses on highly expressive axioms containing various logical connectives and non-taxonomic relation instances. In Figure 1, a simple example illustrates the problem of formal definition generation from unstructured text, along with its important aspects. The figure outlines a typical text mining workflow based on supervised machine learning: data acquisition, feature extraction, training and testing. The workflow is adapted to the task of formal definition generation and contains steps, resources and intermediate states that are needed to extract the formal definition of *“Baritosis”* from its textual definition.

The workflow is not restricted to such textual resources as Web articles or MeSH entries. Input textual definitions can be retrieved from a number of resources, such as encyclopedias and terminologies, PubMed, plugins to known ontology editors (e.g., Dog4Dag [8], that can retrieve textual definitions from the web) and, in principle, any resource that contains textual information relevant for the domain.

The proposed workflow does not fully solve the problem of automatic formal definition generation. However, is seeks to formalize biomedical knowledge in a way that is well established by the life sciences community, i.e., using the same representation as in the SNOMED CT ontology, namely a description logic *EL++* [2]. At the heart of this representation lie the relations that are intersected and existentially quantified. Hence, taxonomic and especially non-taxonomic relation extraction form a very important part of our work. Relation extraction is integrated into a bigger end-to-end pipeline that takes as input biomedical texts and outputs description logic axioms that formalize the information in these texts.

For example, Figure 1 depicts a series of steps, comprising the annotation of the sentence with concepts from a designated ontology, the representation of this textual definition in a feature space and the application of a trained model, e.g., classifier, that has learned to recognize roles (relations) between biomedical concepts, can lead to the final desired output, which is the formal definition of *“Baritosis”*. However, there are three main aspects which comprise the focus of this work and which can give insightful directions on how the task may be addressed efficiently: (a) the *modeling* of the problem, i.e., the selection of the corpora and the relations that may participate in the formal definitions, (b) the *feature engineering*, and, (c) the actual *machine learning* process. Aspect (a) is examined in a setup where the input unstructured text is annotated and then aligned with knowledge about the chosen relations. The analysis of this aspect can illustrate how the definitions mined from different types of corpora influence the final outcome. Aspect (b) aims at examining the importance of different feature types in the learning process. Finally, aspect (c) is meant to provide an insight on the impact that different learning algorithms have, as well as on the number of training examples that are needed per role from the learning process.

## Related work

We start the discussion of the related work with relation extraction. Relation extraction (RE) is the task of detecting and classifying semantic relations that hold between different entities. While it can be performed on both structured and unstructured data, our interest is focused on relation extraction from text.

### Relation extraction for general domain

Textual relation extraction can be performed using different types of linguistic data that one can get from the input text. The most common way is to use the *lexical representation* of the text in order to generate typical patterns for the target relations. The patterns can either be constructed manually [9], or can be leant automatically using machine learning techniques [10].

Certain systems explore the *syntactic structure* of the source text. The motivation behind it is that the semantic relations that hold between the two concepts should be reflected by syntactic dependencies of these concepts. *Learning by Reading* system [11] extracts propositions from syntactic structures of type Subject – Predicate – Object. For the arguments of a relation, i.e. for subjects and objects, the lexical items are generalized to classes (the classes themselves are automatically derived from the corpus). The predicates remain in their lexical form.

Some systems incorporate *semantic information* into the extraction process. The entities and potential relation mentions that have been annotated in the text are assigned more general semantic classes. If a combination of semantic types of the argument concepts and the type of the relation match a certain pattern (which is either induced from an existing ontology, pre-defined manually or appear with a high frequency), the underlying lexical relation is extracted. Flati et al. [12] extract semantic predicates using semantic classes of argument concepts adopted from Wikipedia. Dahab et al. [13] integrate top-level ontologies to semantically parse the input text and to generate semantic patterns of concepts and relations. In the work by Hovy et al. [11] the semantic classes are constructed by the system itself.

With regards to the type of learning that is performed over relations, the task of extracting relations can be done in a supervised way, in an unsupervised way, or in a semi-supervised way, e.g. bootstrapping when an initial seed of relation instances is used. Traditional relation extraction encompasses *supervised learning techniques*. Mohamed et al. [14] state that traditional RE requires “the user to specify information about the relations to be learned”. The information about the relations can be encoded in two ways: (a) for every relation the set of corresponding patterns is manually tailored; (b) relational instances are annotated in the text corpus, and the patterns are acquired explicitly (based on frequent sequences of word tokens) or implicitly (using machine learning). The new relational instances are extracted by pattern-matching or by running a trained machine learning model over the input texts. The supervised approach usually gives high precision of the retrieved relation instances, which can go over 90*%*. This makes supervised learning an ideal technique for tasks that incorporate relation extraction as part of the pipeline and need the RE component to output high-quality relations so that the error would not accumulate throughout the pipeline. This is the reason why our method of generating formal definitions is based on supervised RE.

*Semi-supervised learning* of relations usually has a core of annotated material from which the learning is initialed, and then the process of extraction proceeds in an unsupervised manner. It is ideal for situations when the training data is few. For example, the *NELL* system [15] starts with an initial ontology (a form of prior knowledge) that contains some categories, relations and relational instances. The ontology helps building the first set of patterns that are then used to populate the categories of the ontology and to extract new facts, which are then used to retrain the extraction system and to learn yet new facts etc.

The main distinctive feature of *unsupervised relation extraction systems* is that they do not use any assisting information during learning: they are not provided with the seed examples, or background expressive ontologies, or manually constructed patterns. The learning is performed purely from the input data [11]. One of the popular unsupervised RE approaches is the so-called Open Information Extraction [16]. It is a domain-independent paradigm that uses web-scale-size input corpora of texts. It tends to extract as many triples as possible, but they are not always well-formed or abstract. Both precision and recall of unsupervised RE systems are lower that of the supervised ones. Banko et al. [16] are the pioneers of Open Information Extraction. Their system *TextRunner* works in three steps. First, a deep linguistic analysis is performed over a small corpus of texts. The system itself separates the parsed triples into positive and negative ones. The triples are used for training a machine learning RE model. Secondly, the model classifies the rest of the corpus (millions of sentences) and extracts positive triples. The extraction is done in one pass over the corpus and does not involve the deep processing any more. Lastly, newly extracted triples are assigned a confidence score based on the frequency count of the triple. The system is completely unsupervised, taking raw texts as input and outputting relational triples. Unfortunately, only 1 million out of 11 million high confident triples were evaluated as concrete versus abstract, underspecified facts, e.g. *Einstein – derived – the Theory of Relativity* versus *Einstein – derived – theory*.

### Biomedical relation extraction

The majority of research work on biomedical relation extraction focus on the relations between specific concept types: genes, proteins, diseases and drugs. Heterogeneous pieces of information are mined from various textual sources and assembled together in a form of ontologies, semantic networks, knowledge bases or other knowledge representation structures.

Relation extraction in biomedical domain adopts the methodologies of the general relation extraction. One of the most common approaches is to use lexico-syntactic *patterns*. A set of relevant relations is manually designed by domain experts, and every relation is assigned to a set of textual patterns that are also constructed manually or extracted automatically from texts. Huang et al. [17] extract protein-protein interactions using lexical patterns. Patterns are mined through the dynamic alignment of relevant sentences that mention the interaction. Both the precision and the recall of the system reach 80*%*. Xu and Wang [18] use simple pattern-based approach to extract drug-disease relation instances from MEDLINE abstracts. The patterns are not complicated (e.g. “DRUG-induced DISEASE”), thus the approach exhibits a typical bias towards high precision at the expense of low recall: 90*%* precision and 13*%* recall. However, the majority of extracted instances do not yet exist in a structured way in biomedical databases, which proves the usefulness of the approach. The majority of work on pattern-based relation extraction rely on hand-crafted templates whose construction is a laborious task. In some cases the patterns are built automatically, nevertheless the approach lacks the ability to extract relations that are not explicitly stated in the text, i.e. the relation is not properly mentioned by a verb, a deverbative noun etc, or the two interlinked entities are located to far from each other in the text, and the pattern cannot cover them.

Another common relation extraction approach uses *co-occurrence* information. The idea behind it is quite intuitive: entities occurring in the same sentence significantly often should be related [19]. The drawback of the approach lies in that the correlation information per se cannot capture the type of relation present, i.e. what the formal semantics of the relation is. However, it can efficiently identify potential relations and relation instances that may be examined with other NLP techniques afterwards.

Alternative approach to extract biomedical relations is to use *machine learning techniques*. Firstly, the source text is annotated with biomedical concepts; secondly, sentences or phrases are labeled with relations using external knowledge resources, manual annotation or exploiting the concept types. Finally, a model is trained to discriminate between instances of different classes, i.e. relations. Airola et al. [20] focus on protein-protein interaction extraction and utilize graph kernel based learning algorithm the F score of 56.4*%*. Chun et al. [21] focus on the extraction of gene-disease relations from manually annotated MEDLINE abstracts that describe either pathophysiology, or therapeutic significance of a gene or the use of a gene as a marker for possible diagnosis and disease risks. Incorporating an NER pre-filtering step for gene and disease names the classification performance yields 78.5*%* precision and 87.1*%* recall. Machine learning appears to be a potential approach of relation extraction which does not require to do the tedious work of pattern construction and is able to generalize.

### Formalizing information in textual form

There are several works that attempt to convert textual representation of general knowledge into a structured form. One approach is described in [22]. The authors focus on automatic acquisition of ontology axioms. The formalism of choice is *SHOIN*, an expressive DL that is able to model negation, conjunction, disjunction, and quantitative restrictions. The developed system has several limitations in formalizing the definitional sentences. The majority of limitations stems from the use of hand-crafted rules. In contrast, in our work we attempt to solve this issue by applying machine learning techniques to learn the models of axioms, as shown in Figure 1, which avoid hand-craft patterns on the lexicon or the syntactic structure of a sentence.

An additional related approach that falls into the broad area of ontology acquisition is described in [23]. Given that ontologies consist of terminological axioms (TBox) and assertional facts (ABox), in this paper, we focus on acquiring a special but common TBox knowledge, named formal definitions, from texts. Existing TBox generation approaches are mainly based on syntax-based transformation, but they suffer from the unresolved reference relations (e.g., ∃*Of*) and the lexical variant problems (e.g., “Causative_agent” relation in SNOMED CT can be expressed both by *caused by* and *due to*). Our method is designed to remedy these problems.

To the best of our knowledge, the is no system that does automatic ontology acquisition or definition generation for biomedical concepts. However, in the domain of life sciences there exist several works that move into that direction. A work by R. J. Kate [24] presents the first step towards automated generation of formal definitions for concepts that are not yet present in SNOMED CT. The task is to build a relation identification model that is able to recognize a certain SNOMED CT relation in text. The textual data used in [24] are the clinical phrases from SNOMED CT that describe the concept in natural language (e.g., “acute gastric ulcer with perforation”). A separate machine learning classifier is trained for every typed version of every SNOMED CT relation, e.g., *finding_site(disorder, body_structure)* and *finding_site(finding, body_structure)* yield two separate models. There are three main drawbacks of this work. Firstly, it uses only the data from SNOMED CT clinical phrases, which are formulated in a controlled language. However, the ultimate goal of the system is to be able to identify relations in various medical texts for new biomedical concepts, and these texts are not written in a controlled language. Secondly, the system builds a separate classifier for every relation and its typed version. The resulting system has to run hundreds of models every time a new text passage is processed, which is computationally expensive. Lastly, the work does not discuss how the outputs of multiple classifiers should be combined into a single definition. In our approach we deal with texts of different origin and quality, and we incorporate the information about semantic types of concepts involved in a relation into the feature space instead of training separate classifiers for every combination of concept types and relations.

Okumura et al. [25] automatically process textual descriptions of clinical findings. Every description belong to one of ten categories: anomaly, symptom, examination, physiology etc. Based on the analysis of 161 descriptions, every category was manually assigned a set of typical semantic-syntactic patterns, e.g., a typical way of expressing a pathology is a pattern *substance + verb phrase for phenomenon*, as in *some fibrosis persisted*. The study suggests that there are common ways in which biomedical knowledge is expressed in natural language. Our work uses this finding as one of the motivations to use machine learning techniques and to encode such patterns automatically into models.

Dentler and Cornet [26] eliminate redundant elements in already existing SNOMED CT definitions. Using the ELK reasoner [27], the authors eliminated redundant concepts, existential restrictions and rolegroups. Here is an example of the elimination rule for concepts: if a concept is more general or equivalent to one of the other concepts in the definition of the same concept or a superconcept. This work is highly relevant to the task of formal definition generation, as it provides a method for post-processing that can improve the quality of generated axioms and to make the resulting ontology easier to maintain, construct and expand.

## Methods

Adding new concepts to a formal ontology is a tedious, costly and error-prone process, that needs to be performed manually by specially trained knowledge engineers. By contrast, textual information from the medical domain is widely available from publicly accessible resources, such as the web, textbooks and PubMed articles. In the following we present our methodology towards the automation of formalizing concept definitions from textual ones.

### Problem formulation

Relation instances form the basis of a concept definition; they contain necessary and sufficient information about the taxonomic and non-taxonomic links between the concept to be defined and the other concepts. Table 1 illustrates the connection between a textual definition and its formal representation.

Existing approaches for relation extraction mostly focus on learning superclass or subclass relations [8] (e.g. *Baritosis* - is_a - *Pneumoconiosis* as given in Table 1), leaving out the non-taxonomic relations (e.g. *Baritosis* - caused_by - *Barium*_*dust*). However, the latter are essential for the task of formal definition generation. Existing ontologies in the biomedical domain that contain non-hierarchical relations have the following properties: (1) the set of relations is much smaller than the set of concepts, e.g., SNOMED CT currently has 56 roles, but more than 311,000 concepts, (2) the set of relations remains relatively stable while the concept set is expanded and modified much more often, and, (3) the set of relational instances, i.e. unique semantic links between concepts, is much bigger than the set of relations, e.g., SNOMED CT has more than 1,360,000 relationships.

The observations above suggest that if we are able to extract a relatively small set of relation types, this will result in many relational instances that may populate a knowledge base. Thus, we formulate the problem targeted by the present work as follows: create a system, that for a given set of input texts annotated with biomedical concepts is able: (a) to find text strings that describe a relationship between these concepts, and to recognize, which relationship it is, and (b) to combine these relationship instances into concept definitions. For example, for the target concept *Baritosis* we expect the system to recognize two relations, Causative_agent and Finding_site, from the following two sentences: (1) “Baritosis is a benign type of pneumoconiosis, which is caused by long-term exposure to barium dust”. (2) “Baritosis is due to inorganic dust lies in the lungs”. The corresponding relational instances are: *Baritosis* - Causative_agent - *Barium*_*dust* and *Baritosis* - Finding_site - *Lung*_*structure*.

#### Terminology used

The current work is done on the border of two research areas, namely Text Mining and Description Logic. This section bridges the gap between the terminologies of the two communities, giving equivalent terms to all notions used in the paper.

- **relation**
  In this work we interchangeably use the terms *relation*, *relationship* and *role*. The last term comes from the ontology development research, while the first two terms are used when ontology generation is addressed from the natural language processing viewpoint.
- **triple**
  A binary relation instance is often called a *triple*, since it can be specified by the types of the relation and the two arguments. In linguistic, a triple often refers to a lexical representation of the grammatical structure Subject – Predicate – Object.
- **domain and range**
  Each relation has a *domain* and a *range*, i.e., values for the first and second arguments of the relation, respectively. In linguistic triples, the domain specifies the types of subject and the range specifies the types of object a relation takes.
- **semantic type**
  In this work we define the domain and range of relations using *semantic types*. By them we refer to categories of concepts that can either be *classes* of an ontology (e.g., all the classes of an upper ontology, or several top levels of classes in a an ontology or a taxonomy), or some *concept types* with broad semantics. In the experiments presented in this paper we use three different semantic types: semantic types from UMLS Semantic Network [28], SNOMED CT classes and SNOMED CT categories.

### Learning formal definitions

In the following, we describe analytically the aspects of the suggested methodology towards learning formal definitions from unstructured text.

#### Corpora

Textual corpora and sets of formally defined relations may stem from different sources. The choices are important per se, as to their quality and volume, and in combination with each other. A corpus should adequately represent the domain of choice and should contain necessary and sufficient information about the domain concepts. For the biomedical domain, the following resources are taken into consideration:

**MeSH** (Medical Subject Headings) [29]: Definitions in natural language are produced manually by medical experts and embedded in MeSH, and, thus, are considered precise, scientifically valid, and high quality textual data.

**MEDLINE**: Journal abstracts for biomedical literature are collected from around the world and can be accessed via PubMed [30]. Since MEDLINE contains, among other things, recent publications with cutting-edge advances in biomedicine, it is of particular interest for the task at hand since it enables the formalization and integration of newly emerged biomedical concepts.

**Wikipedia articles**: Wikipedia provides fundamental information about biomedical concepts, which can be easily retrieved by article titles, e.g., *Alastrim*, *Iridodonesis*.

**Web articles**: Besides Wikipedia, many other websites provide relevant knowledge about biomedical concepts^a^. Such information should be filtered from the web pages by selecting sentences of definitional structures. For instance, the Dog4Dag system [8] can retrieve and rank textual definitions from the web.

In this work, we construct the following corpora listed below:

- *MeSH*: Out of 26,853 entries accompanied by textual definitions in MeSH, we selected all concepts that also have definitions in SNOMED CT. For this we used the *UMLS Metathesaurus* [31] which contains mappings of concepts from various knowledge resources, including MeSH and SNOMED CT.
- *SemRep*: Collected from the *SemRep* project that conducted a gold standard annotation study in which 500 sentences were selected from MEDLINE abstracts and manually annotated with 26 semantic relationships [32].
- *WIKI* is obtained by querying Wikipedia with one-word SNOMED CT concept names and amounts to 53,943 distinct sentences with 972,038 words.
- *D4D* contains textual definitions extracted by querying Dog4Dag over concepts that have relationships via the most frequent attributes used for Disease, namely Associated_morphology, Causative_agent, and Finding_site, obtaining 7,092 distinct sentences with 112,886 words.

Unlike the corpus *SemRep*, the other three corpora, i.e., MeSH, *WIKI*, and *D4D* are plain texts without annotations. To use them for learning formal definitions, we developed the alignment process as explained in details in Section ‘Alignment’.

#### Relation sets

Relations in biomedical thesauri are selected and specified by domain experts; therefore we assume that all relations are relevant in terms of semantics, hence they are interesting to be modeled. However, statistically relations are not equally distributed across domain texts; some relations are dominant. For example, for the disease concepts in SNOMED CT, among 48,076 axioms about non-taxonomic relationships, 40,708 of them only use three relations: Associated_morphology, Causative_agent, and Finding_site.

**Figure 2:**
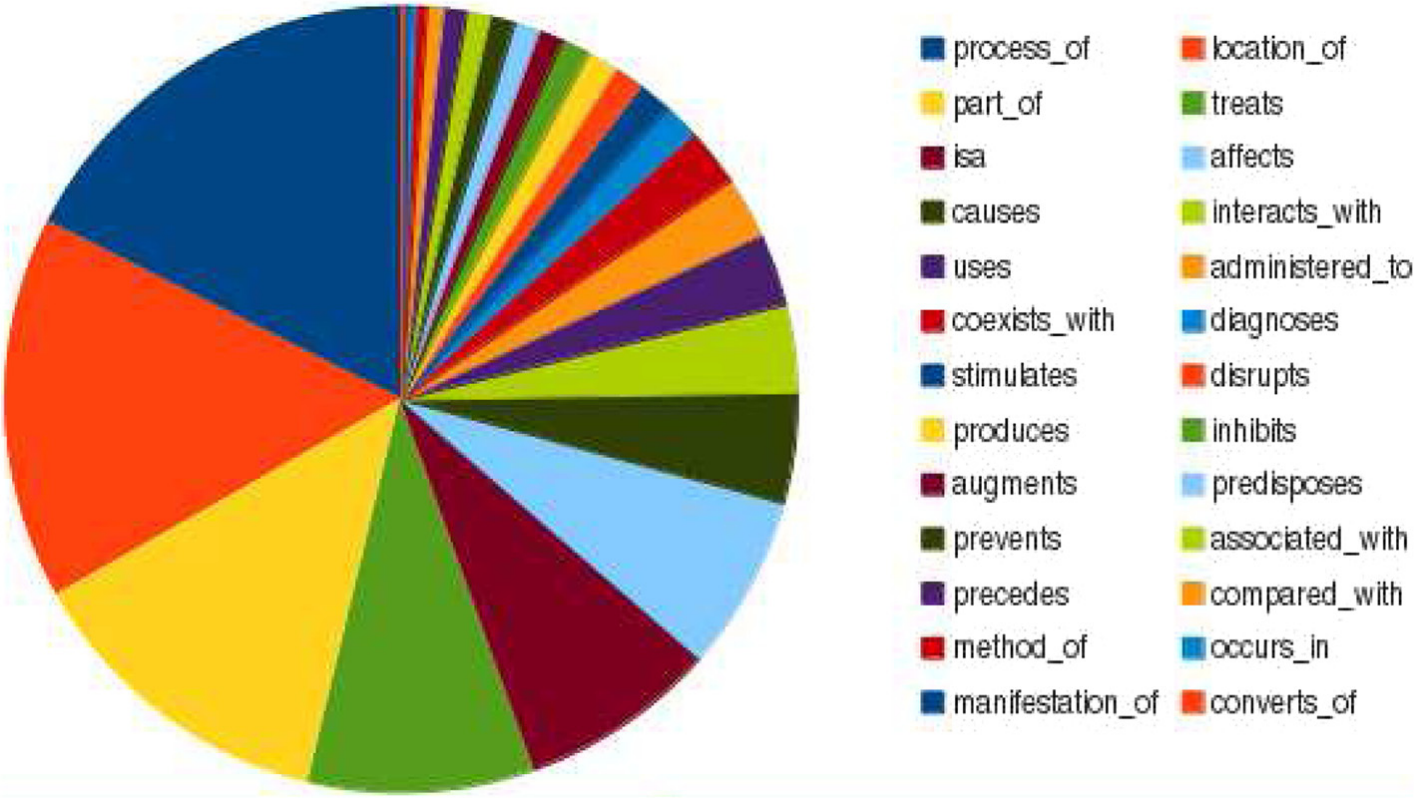
The distribution of relations in the SemRep corpus.

The *SemRep* corpus contains 26 relations, the most frequent ones being Process_of, Location_of, Part_of, Treats, Isa, Affects, Causes etc. The statistical distribution of relations in *SemRep* gold standard corpus is illustrated in Figure 2.

Based on the analysis above, in this work, we focus on two groups of relations:

- The three SNOMED CT relations (Associated_morphology, Causative_agent, Finding_site);
- The 26 relations that occur in the *SemRep* corpus.

Once the relation sets are fixed, we need a set of relation instances to be used as training data. In the case of *SemRep*, we take the instances that are annotated in the corpus. In SNOMED CT, due to its formal semantics, we can distinguish two cases: explicit and inferred relationships. The explicit relationship base (ExpRB) contains all relationships among concepts that are explicitly given in the description of concepts in SNOMED CT. For instance, in Table 1, a human readable display of the formal definition for the concept Baritosis, we have Baritosis|Causative_agent|Barium_dust as an explicit one. The inferred relationship base (InfRB) can be built through a tractable Description Logic (DL) reasoning engine as follows: $\textsf {InfRB} = \{A | R | B : \text {SNOMED CT} \models A \sqsubseteq \exists R. B \},$ where ⊧ is the logical entailment under DL semantics which is tractable for $\mathcal {EL}\text {++}$ [33], the logic language underlying SNOMED CT. By this, we have Baritosis|Causative_agent|Dust as an inferred relationship since Barium_dust is a subclass of Dust by SNOMED CT. By the monotonicity of DL semantics, we have ExpRB⊆InfRB. The details of the two relationship bases for SNOMED CT are summarized in Table 2.

**Table 2:** **Sizes of the explicit and inferred relationships for the relations: Associated_morphology, Causative_agent, and Finding_site**

	**Associated_morphology**	**Causative_agent**	**Finding_site**
InfRB	503,306	91,794	1,306,354
ExpRB	32,454	13,225	43,079

#### Alignment

In our task, a fundamental requirement is the training data from which a model can be learned to recognize formal definitions from texts. When manually annotated corpus is not available, a common case in our experiments, the training data can be automatically created by distant-supervision approach [34]. This consists of two steps: (1) finding the mentions of biomedical concepts in a sentence, and, (2) aligning the sentence with a relation by the following principle: if the sentence contains a pair of concepts, say *A* and *B*, and this pair are arguments of a relation *r* according to a relationship *RB* set under consideration, that is *A*|*r*|*B* is in *RB*, then the sentence fragment between *A* and *B* will be aligned with the relation *r*. This two-step process is illustrated in Table 3. For the given sentence, the fragments *Baritosis* and *barium dust* are the textual mentions of the concepts Baritosis_(disorder) and Barium_Dust_(substance), respectively. By looking up the relationship set, such as ExpRB or InfRB, we know that these two concepts are related by the relation Causative_agent. Thus, the string between these two concepts, i.e. *“is pneumoconiosis caused by”*, is aligned with the relation Causative_agent. Such an alignment process is performed on our MeSH, *WIKI* and *D4D* corpora.

**Table 3:** **Example alignment between sentences and relationships via semantic annotation, and lexical and semantic features extracted from the alignment**

Sentence		“Baritosis is pneumoconiosis caused by barium dust”.					
Annotated Sentence		“*Baritosis* is pneumoconiosis caused by *barium dust*”.					
		Baritosis_(disorder)		Barium_Dust_(substance)			
SNOMED CT relationship		Baritosis_(disorder) | Causative_agent | Barium_Dust_(substance)					
Semantic Features		left type		between-words		right type	
		disorder		“is pneumoconiosis caused by”		organism	
BoW	{is, pneumoconiosis, caused, by}						
Word 2-grams	{is pneumoconiosis, pneumoconiosis caused, caused by}						
Char. 3-grams	{is␣, s␣p, ␣pn, pne, neu, eum, umo, moc, oco, con, oni, nio, ios, osi, sis, is␣, s␣c, ␣ca, cau, aus, use, sed, ed␣, d␣b, ␣by}						

#### Feature engineering

The choice of features is key in classifying relations as it directly influences the success rate of the classification process. To this end, we explore two types of feature: lexical and semantic.

##### Lexical Features:

The lexical features represent specific words, word sequences or word components that link two concepts in a sentence and are located in-between the concept mentions. With regards to the representation of the lexical features we have utilized three approaches: (1) bag-of-words (BOW), (2) word n-grams, and, (3) character n-grams. *BOW* is the most straightforward text representation in which text is viewed as unordered set of words, each unique word in the document collection corresponding to a separate feature. In the *word n-grams representation*, text is represented via all possible word sequences of length up to *n*. Finally, in the *character n-grams representation*, text is represented via all possible character sequences of length up to *n*. All the three types of lexical features separately can be used separately as well as in combination with each other. In Tsatsaronis et al. [10], the results did not prove that the combination of word and character n-grams have a synergy effect on the performance, hence we skip the combination of lexical features in the experiments described below.

The lines in Table 3 starting with “BoW”, “Word n-grams”, and “Char. n-grams” illustrate the lexical features for the definition: “Baritosis is pneumoconiosis caused by barium dust”. The basic assumption behind the choice of features is that each relation has a characteristic way of being expressed in natural language text, which may be captured by the analysis of the words that occur between the two concepts. The values of lexical features, i.e., the three representations of text strings, are binary: the value of a feature is 1, if the corresponding textual element is present in the string, otherwise the value is 0. We have also tried expanding these representations to their weighted versions, assigning real values to features according to their frequencies [10]. However, the weighting scheme of choice turned out to be computationally expensive, but did not yield considerable improvement to the performance. Thus, in the present work we focus on boolean features.

Table 4 gives an example of highly important lexical features for the three SNOMED CT roles, when Word n-grams are used as the feature.

**Table 4:** **Examples of highly weighted lexical features for the three SNOMED CT roles: AM Associated_morphology CA (Causative_agent), and FS (Finding_site)**

*AM*	“displacement of”, “medical condition characterized”
*CA*	“caused”, “cause”, “from the”, “by a”, “agent of”, “an infection of”
*FS*	“of”, “in”, “affects only”, “infection of”

##### Semantic Features:

While lexical features reflect the relation per se, i.e. its semantics and typical ways of expression in the text, semantic features focus on what types of concept arguments a relation can take. They specify the domain and the range of a relation instance. For instance, the relation Finding_site has the subject type Disorder and the object type Body_structure. The motivation behind the use of semantic features is quite intuitive: since every relation has a domain and a range, it can take only certain types of concepts as its arguments. If we include these types into the feature representation of instances, we impose explicit constraints on the arguments of every instance.

Semantic features can help distinguish different relations even though they share some similar lexical features. For example, in Table 4, Causative_agent and Finding_site have similar lexical features “infection of” and “an infection of”, respectively. However, they have different argument types. So for the sentence “Baritosis is an infection of lung”, the relation Finding_site will be recognized instead of Causative_agent, once we know “lung” is of the type Body_structure which is an improper argument type for Causative_agent.

There are several possibilities on how to define a semantic type given a biomedical concept:

- UMLS (grouped) semantic types. The UMLS Semantic Network [35] contains 134 manually built concept types relevant for the biomedical domain. Types are assigned to all the concepts of the UMLS Metathesaurus. However, the modelling of the domain offered by UMLS is not necessarily compatible with that of desired relations, i.e., the types may not fully correspond to the domain and range of relations and, thus, will not form valid patterns of type pairs. Moreover, there are 15 coarser-grained semantic types defined for certain applications, providing a partition of the UMLS Metathesaurus for 99.5*%* of the concepts.
- Upper level classes as types. Another approach is to use the taxonomic structure of a domain ontology. If the taxonomy forms a single tree of concept classes, then the first *n* levels of it can be taken as semantic types. If there are several independent trees, the tree top classes can serve as types. For example, MeSH has 16 taxonomic trees and SNOMED CT has 19 top concepts. They can directly be used as types for their sub-concepts. Indeed, there can be different granularities in choosing a proper taxonomy level as types. However, more fine-grained levels mean more specific information that we know about the target concept, which is often hard to obtain beforehand. Therefore, we consider the level-one top concepts.
- SNOMED CT semantic types. Unlike the top concepts, SNOMED CT has defined semantic types for its concepts which can be read off from the names of the concepts given in parentheses. For example, in Table 4, we have the SNOMED CT concepts Baritosis_(disorder) whose type is disorder and Barium_Dust_(substance) having type substance. Unlike in UMLS Semantic Network, in SNOMED CT a concept has precisely one semantic type.

#### Machine learning

We compared the performance of several classifiers with respect to learning predictive models that can classify new, unseen relation instances. The tested classifiers are: *Logistic Regression* (LR), *Support Vector Machines* (SVM), *Multinomial Naive Bayes* (MNB) and *Random Forests* (RF). SVM yielded the highest performance in our experiments on classifying relations, compared to the other three classifiers. SVM is a linear classification algorithm that automatically builds a hyperplane separating the instances of different classes in such a way that the margin (the distance between the hyperplane and the instances) is maximized.

#### Formal definition generation and evaluation

From relationships discovered from texts, it is easy to traverse to the ${\mathcal {EL}}$ style formal definitions by applying the following transformations to a single (Equation 1) or multiple (Equation 2) relationships, respectively [36]:

(1) $$ A|R|B \rightarrow A\sqsubseteq \exists R.B   $$

(2) $$ \{A|R_{i}|B_{i}\} \rightarrow A\sqsubseteq \sqcap_{i} \exists R_{i}.B_{i}   $$

Besides evaluating the quality of relation extraction, we also evaluate the percentage of candidate definitions that are correct with respect to the formal SNOMED CT definitions. One main problem is that concepts can be defined with multiple ways under the DL semantics. For example we can get a candidate ∃Causative_agent.Dust for the target concept Baritosis. When looking up at the definition given in SNOMED CT, this candidate is not explicitly mentioned. However, this does not affect the definition, since we have ∃Causative_agent.Barium_dust, and because SNOMED CT$\models \textsf {Barium\_dust} \sqsubseteq \textsf {Dust}$ holds, it follows that $\textsf {Baritosis}\sqsubseteq \exists \textsf {Causative\_agent}. \textsf {Dust}$.

The definition precision, *DefPre*, can then be defined as follows, where *Cands*={*A*|*R*|*B*:*A*,*B* are concept names and *Risarelationname*}.

(3) $$  {} \text{\(Def\, \,Pre\)} = \frac{|\{A|R|B \in \text{Cands}: \text{SNOMED CT} \models A|R|B\}|}{|\text{Cands}|},  $$

### Availability of data and software

All corpora used in this work are freely available. MeSH, MEDLINE, SNOMED CT, as well as the UMLS resources (the UMLS Semantic Network, the UMLS Metathesaurus) can be accessed via the official NLM website: http://nlm.nih.gov Annotated corpus from the *SemRep* project (The SemRep Gold Standard corpus) can be obtained from http://skr.nlm.nih.gov/SemRepGold/. The four corpora that were used for training and testing the system, namely *WIKI*, *D4D*, *SemRep* and *MeSH*, are also put in open access in the form of machine-readable *.arff* files and can be found here: http://www.db-net.aueb.gr/gbt/download.html. For the implementation of the machine learning approaches and the representation of the training and test instances in machine format, we used *Weka* (Waikato Environment for Knowledge Analysis), which can be obtained from http://www.cs.waikato.ac.nz/ml/weka/. The implementation of the full pipeline of formal definition generation is published online as a GitHub project: https://github.com/alifahsyamsiyah/learningDL.

### Implementation

For the purposes of the implementation and validation of the suggested approach, we report in the following the technical details with regards to the versions of the resources and tools used. The WIKI corpus was collected from Wikipedia of Nov. 7, 2012, and the D4D corpus was collected using the Dog4Dag plugin on Nov. 9, 2012. With regards to SNOMED CT, the version released as of Jan. 31, 2012 was used. The MeSH hierarchy version used is the official MeSH 2013 release, that was officially released by NLM during December 2012. The UMLS Metathesaurus was used in version 2012AB. The SemRep corpus was last accessed on Sep. 15, 2013. The Weka version used for both training and testing the approach was version 3.6.5. Default settings were used for all of the tested machine learning approaches. In particular, we used Weka implementation of Support Vector Machines, namely of their sequential minimal optimization (*SMO*) version. For all experiments we used the SMO setting with the linear kernel, the complexity parameter C = 1.0 and the epsilon parameter *ε*=1.0*E*−12. The linear kernel can be set up in Weka by choosing the *PolyKernel* kernel with exponent parameter of 1.0. No feature selection was performed. With regards to Metamap, the 2012 version was used, which can be obtained from the following location: http://metamap.nlm.nih.gov/. Default settings were used with options *“-R SNOMEDCT”* to restrict the annotation resource to SNOMED CT.

## Results

We have conducted four different experiments that evaluate the task of formal definition generation in two different levels: (1) learning roles (relations) between concepts, and (2) learning the formal definitions of concepts as a whole. The final definition of a concept consists of relations combined together. Thus, we are interested in evaluating both of these crucial aspects of definition generation, i.e., the way relational instances are formed and the way they are combined into a definition. The first three experiments (Sections ‘Problem formulation’ to ‘Availability of data and software’) account for the level of relations, and the last experiment (Section ‘namerefsec:defpre’) corresponds to the level of definitions.

More precisely, Experiment 1 is an initial attempt to extract biomedical relations from text using machine learning. It explores the potential of different classification algorithms to correctly label instances of three frequent SNOMED CT relationships using lexical features from MeSH definitions.

In Experiment 2 we added a new feature type, namely semantic features, to the learning process and we examined the scenario of learning a bigger set of distinct relations. For this purpose we used the SemRep corpus, that comes with a set of textual definitions manually aligned with 26 relations.

In Experiment 3, we switched the corpus to web-based textual data with the aim to test the robustness of our approach in this setting. The problem of data acquisition is less relevant for Web sources, thus in this experiment we also examined the influence of the data size on the learning performance.

In the last experiment we estimated the quality of generated formal definitions compared to their original forms given by SNOMED CT.

The first three experiments give us insights about all major parameters of the relation extraction process that we outlined in the abstract, i.e., the source of the input corpus, its size, the number of distinct relations and feature representation. Table 5 summarizes the main results of the first three experiments. It shows that with the most important lexical features (character 3-grams) and appropriate semantic types, we achieved F-score larger than 90% on all datasets using 10-fold cross-validation for evaluation. Furthermore, free texts extracted from the Web proved to give a competitive result. One may assume that it is due to the larger data size of the corpus (9,292 v.s. 1,357 or 424 instances).

**Table 5:** **Description of the setup of the three experiments**

	**Modeling**			**Feature engineering**		**Data**	**F-score**	
	**Corpora**	**Relation set**	**Relationship**	**Lexical**	**Semantic**	**Size**	**Without types**	**With types**
**Exp1**	MeSH	SNOMED CT	InfRB	3-grams	—	424	74%	99.1%
**Exp2**	SemRep	SemRep	SemRep	3-grams	UMLS	1,357	51%–54%	94%
**Exp3**	WIKI+D4D	SNOMED CT	InfRB	3-grams	SNOMED CT	9,292	58%–70%	100%

In all experiments Support Vector Machines are used.

The fourth experiment shows that our approach can generate formal definitions with a precision that can reach up to 81%, as it was defined by Equation 3.

### Experiment 1: Lexical features and different classifiers

In the first experiment we used the MeSH corpus described in Section ‘Corpora’ as the source of input texts. We aligned MeSH textual definitions with formal definitions from SNOMED CT ontology (Section ‘Alignment’) and labeled definition substrings with one of the three SNOMED CT relations, i.e., Finding_site, Associated_morphology, Causative_agent. We then converted the textual instances of relations into feature representation using lexical features. We experimented with both word and character n-grams, varying the size parameter *n* from 1 to 4. Then the classification model was trained and tested using 4 different algorithms: Logistic Regression, Support Vector Machines, Multinomial Naive Bayes and Random Forests.

We measured the performance of every combination of features and classification algorithms using the *macro-average F-measure* over the three relations in a 10-fold cross-validation setting. A detailed description and statistics over all settings can be found in [10]. The top performing setting uses character tri-grams and Support Vector Machines, yielding an F-measure of 74%. This result illustrates that the signal lexical features carry is quite strong for the relation classification purposes. However, in order to reach better performance one needs to elaborate on the experiment setting, which was conducted in the subsequent experiments. In the following experiments we report results by using only the SVM classifier setting, since the difference in performance compared to the other classifiers is negligible. The lexical features of choice are character tri-grams.

### Experiment 2: Semantic features and the number of relations

In this experiment we aimed at expanding the relation set from just three SNOMED CT roles to a larger set of diverge, semantically rich relations. The process of aligning MeSH and SNOMED CT definitions provided a dataset of moderate size even for the most frequent relations, and the number of relation instances that we are able to extract via the alignment for less populated relations is insufficient for the automatic learning. Thus we switched to another corpus of definitions, namely SemRep (Section ‘Corpora’).

The SemRep Gold Standard corpus contains both textual definitions and a set of relations and consists of 1,357 relation instances. In addition, we introduced semantic features, reducing every argument concept to its UMLS semantic type (Section ‘Feature engineering’).

We trained and tested the classification models for top 5 and top 10 most frequent SemRep relations as well as for the whole set of 26 relations. The results are given in Table 6.

**Table 6:** **The performance of multi-class relational classifier across three different SemRep datasets**

	**Top 5 relations**	**Top 10 relations**	**All relations**
F-measure			
(with Types)	94%	89.1%	82.7%
F-measure			
(only Types)	93.5%	79.2%	65.5%
Size	860 (63%)	1,144 (84%)	1,357 (100%)

The size of each dataset is specified by the absolute number of instances and by the percentage of instances covered by the respective set of relations. The table reports F-Measure for two settings: including semantic types in the feature space, and excluding them.

As the results show, semantic types seem to offer a big contribution to the overall performance. To answer the question how much do they add to the learning, we repeated the experiment, leaving out semantic features. The results when only n-grams were used, are 54% and 51% for the top 5 and for all SemRep relations, respectively. Compared to the results on the full feature set (94% and 82.7% resp.), the difference in performance rate was 40%. So, semantic types as features are important.

In addition, we examined the effect of the lexical features comparing the results of using both feature types, and of leaving out the lexical features. This is translated into comparing the first and the second line of Table 6. As the results show, the lexical features cannot be neglected as they do offer important contribution in the cases where a relatively large number of relations is considered, e.g., 10 or more.

The second question that we would like to address is whether semantic types are generally effective learning features, or the performance boost was specific to SemRep dataset. For this purpose, we have tried adding semantic features to the Experiment 1, extending the feature representation of MeSH instances with the same semantic types from UMLS. The resulting F-measure of 73.9% is a bit lower than the original one: the semantic types slightly deteriorated the performance, serving as noise to the classifier. However, adding semantic types of different origin had an opposite effect: upper level concepts of SNOMED CT taken as types gave an F-measure of 99.1% for the classification of the three SNOMED CT relations compared to 74.5% with lexical features only. From this we can conclude that semantic types are of great value for the classification, given that their modeling is consistent with the modeling of the relations.

### Experiment 3: Web corpora and the dataset size

The third experiment introduces Web-based corpora as the source of textual definitions from which formal definitions and relation instances are built. We used two different corpora, WIKI and D4D, automatically extracted from the Web and analyzed the impact of corpora for our task. The results are reported in Table 7. In accordance to the findings of the previous experiment, semantic types (the SNOMED CT types) improved the results notably, achieving an F-measure of 100% on both corpora. This is largely due to the disjointness of argument types for the three target roles from SNOMED CT. Moreover, Table 7 shows that the use of D4D always improved the final performance compared to the use of WIKI. An explanation for this can be that the sentences from D4D are filtered beforehand by syntactical patterns [8].

**Table 7:** **Main results on the web corpora WIKI and D4D, where the lexical feature is character 3-grams and type is the SNOMED CT semantic type as discussion in Section ‘** Feature engineering’

	**Char 3-gram**	**Char 3-gram + Type**
WIKI	58%	100%
D4D	70%	100%

Overall, WIKI and D4D corpora are much larger that SemRrep and MeSH corpora (Section ‘Corpora’). In fact, having a dataset of more than 60 thousand sentences, it is possible to plot the performance of the relation classification as a function of the dataset size and to analyze the impact that the number of learning instances has on the learning. Using the settings of Experiment 3, we discuss this impact in Section ‘Does data size matter?’.

### Quality of generated formal definitions

The final step is to translate the predicted relationships into formal definitions. For this, we consider SNOMED CT as a reference ontology, which has ${\mathcal {EL}}$ as the underlying logical representation, and, hence, no subjective judgement is involved in the analysis. In particular, we consider the concepts that are descendants of Disease (disorder). Although there is a total of 65,073 descendants of Disease, not all of them have textual mentions in the corpora we studied. We examined 1,721 concepts, such as Contact_dermatitis and Subdural_intracranial_hematoma, which have occurrences in the collection of WIKI and D4D corpora, according to Metamap. For these 1,721 concepts, we obtained an average precision of 66.5*%* (defined by Equation 3). The low value is due to the fact that there are 314 concepts that occur in the sentences which do not contain suitable information for extracting their formal definitions, thus, obtaining zero precision. Considering merely the remaining 1,407 concepts, we achieved a precision of 81.3*%*.

We further investigated if formal reasoning can be helpful for our task. For this, instead of using InfRB as done in all other experiments, we use ExpRB, as given in Table 2, to construct training data. Note that ExpRB is a proper subset of InfRB, so less training data can be obtained in this setting compared to using InfRB. As a result, the average precision of definitions decreased to 61.4% from 66.5% for the 1,721 concepts. The 5.1 p.p. precision difference shows that the dataset automatically enriched by formal reasoning (the use of InfRB) improves the system’s quality in predicting formal definitions of SNOMED CT concepts. This is because the inferred relationship base brings more training examples to boost the whole learning procedure.

## Discussion

The above experiments show that automated conversion of textual definitions into formal ones is a hard, but feasible problem. In this section we briefly discuss the choice of corpora, lexical and semantic features, learning algorithms and data size and how they influence the performance of the definition generation pipeline. Sections ‘Do corpora matter?’ to ‘Does data size matter?’ directly correspond to the questions we posed at the beginning of the paper, and Section ‘Chosen formalism’ sets an open question of which logic better suits the task of formal definition generation.

### Do corpora matter?

At a first glance, for extracting formal definitions from texts, the textual data should have a big effect on the system. For example, MeSH contains manually edited textual definitions for concepts, which should be of an obvious advantage for this task. However, from Experiment 3 and 4, the experiments on automatically extracted web corpora WIKI and D4D, we can still achieve formal definitions of a good quality based on both F-score and the definition precision. Set aside MeSH, WIKI and SemRep corpora, which are manually curated, this might be explained by two facts: (1) the alignment process, described in Section ‘Alignment’, does ensure that the aligned examples are descriptive of the target relation set, and, (2) the D4D plugin prioritizes the definitional sentences from the Web, giving higher scores to trustworthy resources. Overall, the selection of the corpus given the four choices, did not affect much the final performance, with the approach providing good generated definitions in all cases.

### Does feature representation matter?

#### Lexical features

We have tried two different lexical features, word and character n-grams, of various size of up to *n*=4 (word unigrams constituting the basic *bag of words* model). Since the character n-grams model consistently outperformed the word n-grams model in the Experiment 1, given the same value of *n*, in the subsequent experiments we focused on the use of characters. The n-gram model incorporates as features all possible combinations of characters of length *n* that are present in the input corpus, hence the size of the model is exponential to *n*. For this reason the goal is to find the minimum value of the parameter *n* that yields one of the highest performance. In Experiment 1 we noticed that character 3-grams perform almost as good the 4-grams while keeping the size of the model computationally feasible [10]. The expansion of the model to 5-grams and beyond is thus unnecessary. While uni-grams and bi-grams do not convey information that is statistically relevant for the classification, 3-grams, in contrast, are able to capture stems of the key words, important morphemes, word order etc. They are shallow linguistic features that are easy to generate and integrate into the model, they lead to the top classification performance among other lexical features and are used in all experiments. Finally, in Experiment 2 we showed that, although semantic types as features had the biggest influence on the performance, lexical features are still of high value in cases where the target relation set is relatively large.

#### Semantic features

Semantic features represent broad categories of concepts serving as relation arguments. They are the features that drastically influence the classification performance. Semantic types impose category constraints on the argument values of the relation. Taken together, all instances of the same relation form typical patterns of semantic type pairs that are specific for this particular relation. As features, semantic types convey a strong signal to the classifier which relation to choose. The discriminative power of semantic types of relation arguments is the strongest if the patterns for different relations do not overlap. The more semantic type pairs are shared by more than one relation, and the more instances are covered by those pairs, the more erroneous is the classification based on these features. In Table 6 we can see that the performance slowly deteriorates with the growing number of distinct relations. In fact, taken as the only features, semantic types yield lower results than in combination with lexical features for the number of relations of ten and more. Thus, the combination of lexical and semantic features is utilized for such cases.

#### How to choose the right semantic types?

Experiments 2 and 3 illustrated that involving semantic types gave a great boost to the system. In our approach to formalizing textual definitions, we selected predefined relations from existing biomedical thesaurus, such as UMLS and SNOMED. Note that each thesaurus has a different way to define the semantic type for a concept [37]. As in Experiment 2, the UMLS semantic types were used for SemRep relations, and in Experiment 3, the SNOMED CT types were used for SNOMED CT relations. A natural question would be if the consistency between relations and semantic types matters. To this end, we performed an experiment on MeSH corpus with SNOMED CT relations but with consistent (SNOMED CT) and inconsistent (UMLS) semantic types, respectively, as features. We achieved 99% F-measure in the consistency case, and only 74% for the inconsistency one. Compared to the baseline 75%, inconsistent matching did not improve, instead, it even weakened the system. This illustrates that the constraints imposed by SNOMED CT types are unambiguous for SNOMED CT relations, but overlapping according to UMLS types.

#### Example of the influence of semantic types

To examine thoroughly the effectiveness of the semantic type feature for predicting relations, let us recall Table 4 which contains similar lexical features for two different relations: *“an infection of”* for the Causative_agent relation, and *“infection of”* for the Finding_site relation. Indeed, the string *“an infection of virus”* denotes the existence of a causative agent relation, while the string *“infection of stomach”* gives the location of a disease, and, thus, it denotes the existence of a finding site relation. Because the two strings are practically the same, i.e., *“infection of”*, by examining only the lexical information in this case it is hard to decide which relations should be assigned to the two strings. However, Causative_agent and Finding_site have specific combinations of semantic types with regards to the domain and range. As its range argument, Causative_agent relation may have a concept of the semantic type Organism. The Finding_site relation may have Body_structure. In parallel, Causative_agent cannot have Body_structure as a range argument, and Finding_site cannot have Organism as its range argument. Hence, since *“virus”* has Organism as its semantic type, and *“Stomach”* has Body_structure, the machine learner can easily assign correct relations for the strings *“an infection of virus”* and *“infection of stomach”*. Therefore, in this case our approach identifies a Causative_agent relation in the former case, and a Finding_site relation in the latter case.

### Do ML algorithms matter?

The choice of the machine learning algorithm is secondary for the task at hand. While in [10] Support Vector Machines consistently dominate over Logistic Regression, Random Forests and Multinomial Naïve Bayes, the difference in performance rate values are partly due to the small size of the dataset (424 instances). In several cases, SVM have been shown to match or dominate the performance of competitive techniques for major text mining exercises. For example, in [38] it is shown that SVM, Naïve Bayes and k-Nearest Neighbor are among the best performing classifiers. A later work by Mladenić et al. [39] evaluates several classifiers on the Reuters news datasets showing that SVM tend to outperform other algorithms including Naïve Bayes.

However, several studies on the use of Big Data suggest that the performance rates of various algorithms tend to converge given considerable amount of instances. In [40], in the task of word sense disambiguation, the performance of several algorithms (Naïve Bayes, Winnow, Perceptron) gradually increases and eventually converges as the training dataset size increases. Colas and Brazdil [41] conclude that, although SVM are among the most effective algorithms in the area of text processing, various other algorithms, e.g., Naïve Bayes or k-NN, achieve comparable performance. Hence, in our work we shift the focus towards feature engineering and use only SVM.

### Does data size matter?

For the experiments introduced in Section ‘Results’, the corpora data sizes moved from 424 to 9,292, on which we achieved consistent results as analyzed above. Indeed, we conducted similar conclusions by sampling smaller data sets from the large corpora WIKI and D4D: (a) The performance increased with the increase of the data size if only lexical features were used: on D4D, F-score ranged from 59% (data size 300–500) to 63% (data size 750–1,000), and to 70% (data size 1,500–3,100). On WIKI, F-score ranged from 48% (data size 600–700) to 57% (data size 800–3,000), and to 67% (data size 4,000–6,000). (b) The performance stayed relatively constant if semantic features were involved too, no matter the size of the data: F-score fell into the range of 98% to 100% on all the sampled smaller data sets. This again confirms that semantic type is a key feature for the task.

### Chosen formalism

In this work, we choose to learn ${\mathcal {EL}}$ style biomedical ontologies, adopted by SNOMED CT, for the following reasons: (a) the results can be directly compatible with and integrated into the existing resources, (b) it is possible to evaluate our work, using SNOMED CT as a benchmark, (c) the generated definitions can be easily manipulated by users who have previous experience with SNOMED CT, (d) ${\mathcal {EL}}$ can deduce implicit hierarchies among the SNOMED CT concepts.

However, there are many other Description Logic styles that might be interesting to consider, for example, the full version of GALEN [42] which was designed to be a re-usable application-independent and language-independent model of medical concepts. This opens our future work on extending the proposed method for learning more expressive biomedical ontologies.

### What kinds of definitions are generated by the method?

${\mathcal {EL}}$ imposes strict constraints on the form of the definitions that are generated:

- The left part is the concept to be defined, and the right part is the intersection (conjunction) of existentially quantified (restricted) roles. The two parts can be linked into an axiom either by an equivalence operator ≡ (making it a DL definition in the strict sense), or by a subsumption (inclusion) operator $\sqsubseteq $ (thus making it a primitive definition, that only specifies the necessary condition). In the current work we make all generated definitions to contain a subsumption operator.
  Ex: *Arthritis is a form of joint disorder that results from joint inflammation.*
  $\mathsf {Arthritis} \sqsubseteq \mathsf {Joint\_Disorder} \sqcap \exists \mathsf {results\_from}. \mathsf {Joint}\mathsf {\_Inflammation}$
- The definition cannot contain negation or disjunction of concepts, cardinality constraints, universal quantification etc., as these are not part of the $\mathcal {EL}$ syntax.
- The definitions have flat structure. If there is an existential restriction of a concept in the right part of the definition, then this concept should be a simple concept and not of the form C_1_⊓C_2_ or ∃R_1_.C_1_.

This, however, does not by any means prevent the definition from containing several relations, which is often the case for biomedical concepts, as it was rightfully stated by the reviewer.

(4) $$\begin{array}{*{20}l} {\fontsize{6.1pt}{9.3pt}\selectfont{\textsf{Fox-Fordyce\_Disease} \sqsubseteq \exists \textsf{Finding\_site}. \textsf{Apocrine\_glands} \sqcap}} \\ {\fontsize{6.1pt}{9.3pt}\selectfont{\exists \textsf{Finding\_site}. \textsf{Intraepidermal\_apocrine\_ducts}}} \sqcap \end{array} $$

(5) $$\begin{array}{*{20}l} {\fontsize{6.1pt}{9.3pt}\selectfont{\exists \textsf{Causative\_agent}. \textsf{Obstructure} \sqcap}} \end{array} $$

(6) $$\begin{array}{*{20}l} {\fontsize{6.1pt}{9.3pt}\selectfont{\exists \textsf{Causative\_agent}. \textsf{Rupture} \sqcap}} \end{array} $$

(7) $$\begin{array}{*{20}l} {\fontsize{6.1pt}{9.3pt}\selectfont{\exists \textsf{Associative\_morphology}. \textsf{Papular\_eruptions}}} \end{array} $$

## Conclusion

In this work we explored the problem of formal definition generation from textual definitions of biomedical concepts. We suggested a machine learning based method that automatically generates Description Logic axioms from textual data. At the core of the method lies the relation extraction component. Once formal relation instances are generated from text, they are combined into definitions. The method is implemented and is made available to the public. We tested the method on three benchmark data, evaluating it both at the level of relations and at the level of definitions and achieving a success rate of over 90*%* and 80*%*, respectively. Moreover, we investigated in detail how different aspects of the method, e.g., the source of textual definitions or the types of features used for learning, affect its performance. Overall, the choice of corpora, lexical features, learning algorithm and data size do not impact the performance as strongly as semantic types do. Semantic types limit the domain and range of a predicted relation, and as long as relations’ domain and range pairs do not overlap, this information is most valuable in prediction. Our work demonstrated that automated conversion of textual definitions into formal ones is a hard, but feasible problem. It enables complex reasoning over biomedical knowledge that still exists only in unstructured textual form and can contribute to many biomedical applications.

## Endnote

^a^ E.g., http://www.primehealthchannel.com or http://topdefinitions.com
